# Video Chat Technology to Support Home and Community-Based Organizations

**DOI:** 10.1093/geroni/igab046.1646

**Published:** 2021-12-17

**Authors:** Madina Khamzina, Brielle Ross, Allura Lothary, Dillon Myer, Raksha Mudar, Wendy Rogers

**Affiliations:** 1 University of Illinois Urbana-Champaign, Champaign, Illinois, United States; 2 University of Illinois Urbana-Champaign, Urbana, Illinois, United States; 3 OneClick.Chat, Philadelphia, Pennsylvania, United States; 4 University of Illinois-Urbana Champaign, Champaign, Illinois, United States

## Abstract

Concerns about loneliness and social isolation for older adults were already evident but have been exacerbated during the pandemic. Home and Community Based Organizations (HCBOs) provide support for their older clients in the community and need to support their staff, who may be working remotely. We are exploring the potential of video chat technology to connect older adults with their friends, families, and other support. We review the technologies available to older adults in the community and staff working with older adults to promote social engagement. We are collaborating with OneClick.chat to identify the needs of the HCBOs through a literature review and qualitative interviews of staff members from different senior living environments. Their challenges and successes of engaging older adults through video chat technologies will provide guidance for design of an HCBO dashboard for OneClick.chat that will support diverse needs.

